# Photopic flicker optoretinography captures the light-driven length modulation of photoreceptors during phototransduction

**DOI:** 10.1073/pnas.2421722122

**Published:** 2025-02-13

**Authors:** Sławomir Tomczewski, Andrea Curatolo, Andrzej Foik, Piotr Węgrzyn, Bartłomiej Bałamut, Maciej Wielgo, Wiktor Kulesza, Anna Galińska, Katarzyna Kordecka, Sahil Gulati, Humberto Fernandes, Krzysztof Palczewski, Maciej Wojtkowski

**Affiliations:** ^a^International Centre for Translational Eye Research, Warsaw 01-230, Poland; ^b^Institute of Physical Chemistry, Polish Academy of Sciences, Warsaw 01-224, Poland; ^c^Department of Physics, Politecnico di Milano, Milan 20133, Italy; ^d^Faculty of Physics, University of Warsaw, Warsaw 02-093, Poland; ^e^Gatan, Inc., Pleasanton, CA 94588; ^f^Center for Translational Vision Research, Department of Ophthalmology, Gavin Herbert Eye Institute, University of California, Irvine, CA 92697; ^g^Department of Physiology and Biophysics, University of California, Irvine, CA 92697; ^h^Department of Chemistry, University of California, Irvine, CA 92697; ^i^Department of Molecular Biology and Biochemistry, University of California, Irvine, CA 92697

**Keywords:** photoreceptors, phosphodiesterase 6, sildenafil, optoretinography, functional retinal imaging

## Abstract

In this study, we used an inhibitor of phosphodiesterase 6 (PDE6) to examine the impact of changes in the conformation of the PDE6 protein on the light-induced process responsible for altering the length of the outer segments of photoreceptor cells in both human and rodent eyes. We employed a imaging method called spatiotemporal optical coherence tomography, which ensures high contrast and phase stability within the strongly scattering photoreceptor– Retinal Pigment Epithelium complex. Using this approach, we recorded nanometer-scale changes in human cones and rods in response to photopic flicker stimulation and observed length changes in rodent rods under scotopic conditions following a single pulse of light, in the absence or presence of sildenafil, which inhibits the catalytic activity of PDE6. Our findings are consistent with the interpretation that during phototransduction conformational changes in PDE6 structure, which occur on an angstrom scale, are amplified to the nanometer scale due to the unique structure of the photoreceptor outer segments and sequential stimulation. This finding opens up possibilities for the informed use of photopic flicker optoretinography measurements as a diagnostic tool, as the observed nanometer-scale changes in rod and cone dimensions as a function of light stimulus can now be directly linked to molecular events involved in the phototransduction pathway.

## Significance

Assessing the efficacy of state-of-the-art treatments of eye diseases necessitates the development of - tools to monitor retinal function in a noninvasive, noncontact, rapid, and quantitative manner. This study documents the biochemical mechanism responsible for the nanometer-scale elongation of the retinal photoreceptor outer segments (POS) in response to light stimulation. To measure this molecular-level change, we developed a method using a novel spatiotemporal optical coherence tomography approach with photopic flicker excitation, termed flicker optoretinography. The elongation of the outer segments is likely driven by conformational changes in the phosphodiesterase 6 protein during phototransduction. These processes are amplified from the angstrom molecular scale to the nanometer cellular scale due to the unique structure of the POS.

One of the ongoing challenges in assessing vision loss and evaluating the efficacy of potential retinal therapies is the development of a functional imaging technique that is noninvasive, contact-free, fast, and quantitative. As light induces physical changes in different layers of the retina, designing the next generation of functional imaging techniques requires the identification of a biological mechanism that links measured physical-dimensional values to molecular-level events that mediate the visual process.

Early observations of photoreceptor physical changes date back to studies by Korenbrot and Cone (1, 2) and Bownds and Brodie (3), which documented light-sensitive swelling of isolated frog rod outer segments (ROS) due to changes in cation flux and concomitant osmotic pressure changes (1–3). Subsequent X-ray diffraction studies revealed light-induced disc swelling and changes in the spacing between discs in isolated ROS (4). Throughout the 1980s and 1990s, optical methods were also developed to track subtle changes in brain reflectance caused by membrane depolarization and cellular swelling (5, 6). In eye research, fundus reflectometry has been used to measure changes in light reflectance in response to stimulus-light patterns at different wavelengths. An infrared fundus camera system equipped with a charge-coupled device camera could noninvasively detect rapid cone- or rod-induced reflectance changes in the macaque retina (7). These results correlated with multifocal electroretinography (ERG) responses at corresponding retinal locations (7) and paralleled findings on optical transmission changes observed in frog and bovine retinas (8–10). Studies with cats demonstrated that changes in near-infrared (NIR) reflectance signals on a timescale of single seconds were confined to the stimulated region, and they were attributed to the hemodynamic component of the retina’s intrinsic optical signals (11), building on earlier studies of intracellular responses in the same animal model (12).

In vivo measurements predominantly utilize optical imaging methods, with optical coherence tomography (OCT) leading the way. In 1994, Mulligan proposed the term “optoretinogram” (13), a theoretical optical equivalent to the signal obtained using the well-established ERG method. Over a decade later, three independent groups used OCT to capture such optical signals, originating from animal retinas in vitro and in vivo, manifested as changes in retinal layer reflectivity in response to a single pulse of visible light (14–16). Around the same time, changes in reflectivity from individual photoreceptors were recorded in humans in vivo using adaptive optics-equipped systems, including a flood-illumination retina camera (17) and a scanning laser ophthalmoscope (18).

In 2010, it was demonstrated that OCT could detect responses to flickering light, similar to flicker ERG (19, 20). Later, studies showed that optoretinograms could be obtained by measuring phase differences in OCT volumes before and after illumination (21). In 2021, optoretinography (ORG) was shown to detect decreased responses in retinitis pigmentosa subjects, even in areas where cone density appeared normal (22). The following year, ORG signals were reported based on changes in the inner plexiform layer (IPL) in response to flickering light (23).

In another study, visible light stimulation over a 200-fold intensity range caused ROS elongation and increased light scattering in wild-type mice, but not in mice lacking the rod G-protein alpha subunit, transducin (Gαt), indicating that these responses were triggered by phototransduction (24). However, the increased backscattering from the ellipsoid zone of the OS, called the IS/OS junction, can be explained by a model combining cytoplasmic swelling, translocation of dissociated G-protein subunits from the disc membranes into the cytoplasm, and relatively higher H_2_O permeability of nascent discs near the ellipsoid zone of the ROS. Moreover, the slow rise of the signal was incompatible with the time course of phototransduction.

**Fig. 1.**
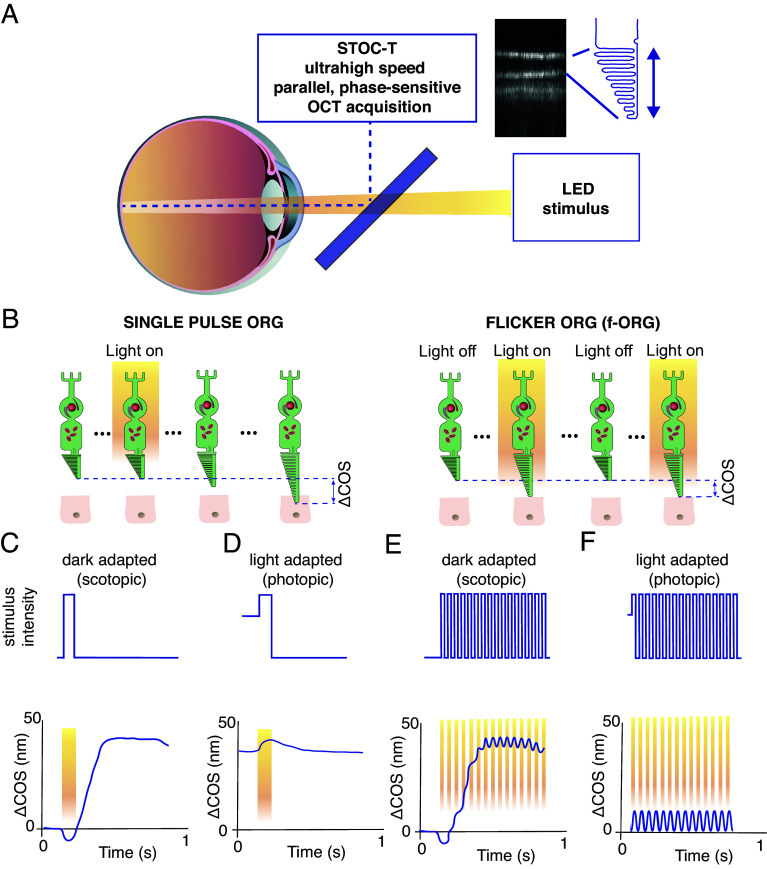
Schematic diagram of cone-mediated ORG in the human eye—measurement strategies. (*A*) A simplified schematic of the STOC-T ORG system, which allows high-contrast volumetric reconstruction of the STOC-T retina to be achieved within 5.32 ms. ORG measurements require 340 repetitions in 1.8 s, allowing stable registration of the phase difference of the STOC-T signal between the backscattering layers (IS/OS and COST). (*B*) Light stimulation can be implemented under scotopic and photopic conditions using a single pulse or flickering light. (*C*) When stimulated by a single pulse under dark-adapted conditions, cones change the length of their outer segments in a time frame of seconds. (*D*) Under photopic conditions, single pulse stimulation results in a smaller change in the length of the COS. (*E*) With flickering light stimulation (f-ORG), cones periodically change the length of their outer segments (∆COS) within tens of milliseconds, with an amplitude that is an order of magnitude smaller than that with single-pulse stimulation. (*F*) When stimulated under photopic conditions, oscillatory ∆COS is detectable for f-ORG without a superposed slow slope curve.

Later work demonstrated that light-induced optical-phase changes occur in cone cells (25). It was suggested that these phase dynamics arise from physiological activity occurring on dramatically different timescales (from milliseconds to seconds) inside the cone outer segment (COS), encompassing the phototransduction cascade and subsequent downstream structural effects. Recently, ORG signals have been measured from various layers of the rat retina, including the inner and outer segments of rod photoreceptors, the retinal pigment epithelium, and the subretinal space (26). ORG has also been applied successfully in humans for photoreceptor classification (22, 25, 27) and in mice for measuring diurnal variations in ROS length (28) and dark-adaptation dynamics (29). We have recently implemented ORG with a new variant of the OCT technique called spatiotemporal optical coherence tomography (STOC-T). Its high acquisition speed in full-field mode, reduced cross-talk, and improved phase sensitivity together enable us to record nanometer-scale changes in photoreceptor length (Fig. 1*A*) and capture ORG signals for single-pulse and flicker stimulation in vivo over a broad range of frequencies (30–34).

So far, in vivo ORG experiments with humans have mostly investigated the effect that a single flash of light has on the COS under conditions of dark adaptation (single-pulse dark-adapted ORG) (Fig. 1*B*) (21, 25, 35–37). After a brief contraction of about 5 ms, the optical path length of the COS (ΔCOS) expands at a rate of hundreds of nanometers per second (Fig. 1*C*) (21, 25, 35–37). Pandiyan et al. explained the initial fast OS shrinkage as the optical manifestation of electrical activity caused by changes in electrical potential and surface tension within the photoreceptor disc membranes (35). It has also been shown that the cone elongation responses are characterized by the sum of two exponentially increasing components that differ more than 10-fold in time constants, and fourfold in light sensitivity (37).

In the present study, our experimental design and results have implicated the involvement of the phototransduction cascade in the morphological changes observed in the photoreceptor outer segments (POS) under both a single-pulse stimulus or flickering light stimulus, known as ORG. One observed effect is the recently described elongation of the POS in response to varying light stimuli (21, 25, 33, 35–41). We hypothesize that the light-driven molecular conformational change of PDE6, identified as a pillar protein between both types of photoreceptor OS disc membranes, plays a primary role in mediating the relatively rapid bidirectional change in POS length (i.e., elongation and shrinkage) synchronized with the modulation of the flickering-light stimulus luminance. We further hypothesize that the same mechanism applies to both cones and rods, albeit with different overall kinetics, accordingly this mechanism would be implicated in both photopic, cone-mediated responses to a flickering stimulus and scotopic, rod-mediated responses to a single flash stimulus, even across species (e.g., in the retinas of both primates and rodents). By relating human f-ORG and single-pulse ORG in mice with drug-inhibited phototransduction responses, we obtained evidence to explain the molecular basis for the changes observed in the f-ORG signal.

## Results

### Cone-Mediated flicker ORG in the Human Eye Imaged In Vivo.

**Fig. 2.**
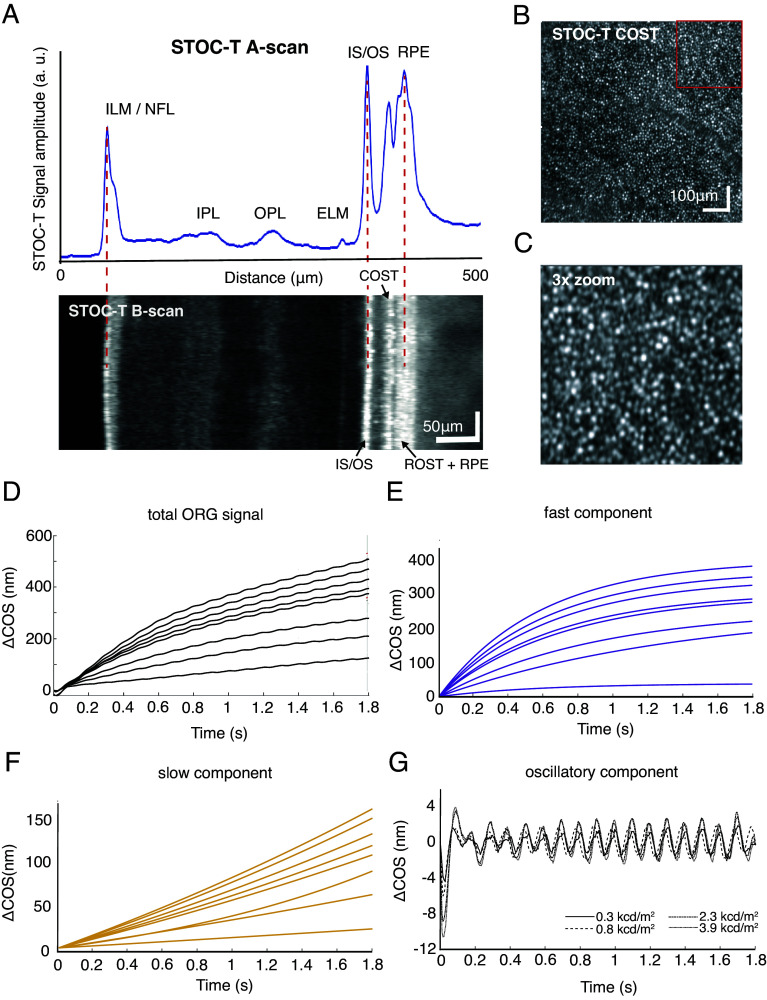
Scotopic cone-mediated flicker ORG (f-ORG) in the human eye in vivo. (*A*) Results of STOC-T imaging with a representative A-scan and B-scan of the retina in a 0.5 × 0.8 mm area (Z × X). COST, cone outer segment tips; ROST, rod outer segment tips. (*B*) Representative en-face STOC-T image from the depth corresponding to the cone tips, showing the number of cones from which signals are averaged for f-ORG. (*C*) 3 × enlargement of the en-face STOC-T image of the cone mosaic—the region corresponding to the red square in panel *B*. (*D*) Dark-adapted responses to the 10-Hz flicker stimulus, recorded with variable peak-stimulus illuminance recorded between 0.3 and 3.9 kcd/m^2^ (1.7 to 18.5% of bleach). (*E*) Fast component decomposed from the data presented in panel *D*. (*F*) Slow component decomposed from the data presented in panel *D*. (*G*) The oscillatory component as a time-varying signal; the amplitude of the oscillations changes slightly with changes in the luminance of the stimulating light due to partial depletion of the photopigment during the flicker.

A schematic diagram of cone-mediated ORG in the human eye is presented in Fig. 1, along with an outline of the measurement strategy that is elaborated under *Materials and Methods*. The outer segments of photoreceptors in association with the RPE form three clearly distinguishable hyperreflective layers in B-scan OCT sections (Fig. 2*A*). One of these layers corresponds to the location of the cone outer segment tips (COST). A coronal projection of this layer (en-face image) reveals a well-defined mosaic of cones (Fig. 2*B*), facilitating the counting of local cone density in the stimulated area when zoomed (Fig. 2*C*). The dark-adapted eye (scotopic conditions) experiences a change in COS length when stimulated by a flickering light over time (Fig. 1*E*), with a small, oscillatory contribution (Fig. 2*D*). This harmonic signal, with an amplitude of approximately a few nanometers (~2 nm for a 10-Hz stimulus), is superimposed on the slower-changing COS-elongation signal known from previous publications (21, 24, 37).

Similar to the findings of Pandiyan et al., the COS responses to stimulating light under scotopic conditions can be accurately described as comprising two exponentially increasing components that differ by about tenfold in terms of time constants (37). To separate and visualize these two components, we fitted each response trace using a weighted sum of two exponentially increasing functions. Subtracting one component from the original traces enabled us to visualize the individual components, which we identified as the fast component and the slow component (Fig. 2 *E* and *F*). This decomposition reconstructed the response set, with variations of the oscillatory component (Fig. 2*G*), and noise.

**Fig. 3.**
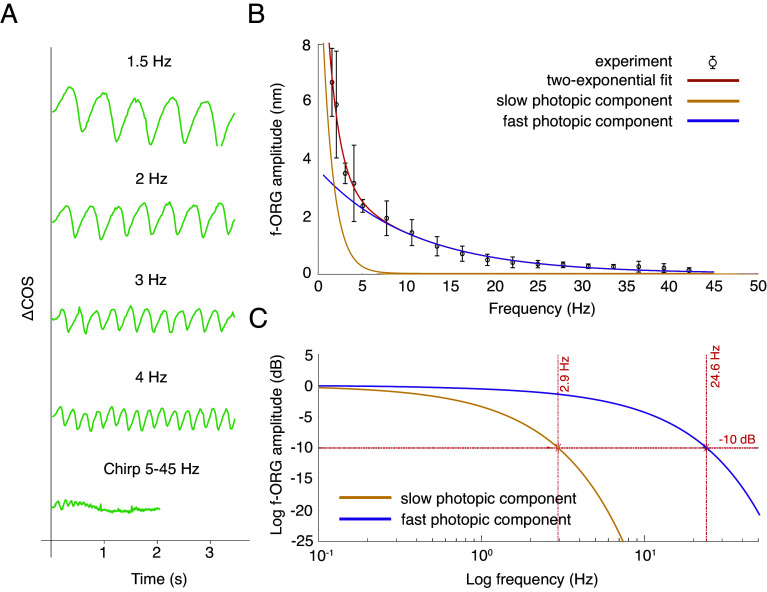
Photopic cone-mediated flicker ORG in the human eye in vivo. (*A*) Examples of readouts of the cone-response signals for different stimulus frequencies. (*B*) Plot of the change in amplitude of the f-ORG signal as a function of stimulus frequency, along with a two-exponential fit indicating the two components of the observed signals. The average frequency characteristic for all subjects is presented as black circles, with vertical bars representing one SD between individual responses to flickering light at each frequency. (*C*) Two components describing the f-ORG frequency characteristics of the cones presented on a logarithmic scale with 2.9 Hz and 24.6 Hz cut-off frequencies at −10 dB, and time constants of 398 ms and 43 ms, respectively, measured for a signal drop of −3 dB (for clarity, not marked on the plot); analysis derived from data obtained from six volunteers (66 ORG signals in total).

In the subsequent experiments conducted on the human eye in vivo, we focused exclusively on measuring the oscillatory component of the photoreceptor response to the flicker stimulus. To isolate the oscillatory part of the signal, the retina was light-adapted (photopic conditions) for 1 min before each data acquisition, after which the flickering light stimulus was applied (Fig. 1*F*). To thoroughly investigate the elongation of the POS, we measured the oscillatory responses across a frequency range of 1.5 to 45 Hz. For the low-frequency band (1.5 to 5 Hz), we used a sequence of 4 separate frequencies (33), while the response at higher frequencies (5 to 45 Hz) was measured using chirped stimuli (34). Representative f-ORG signals recorded from a single measurement in one subject are shown in Fig. 3*A*. For each subject, we aimed to acquire at least three measurements for each frequency and chirp. The final number of successful ORG measurements used to create the plots presented in Fig. 3 *B* and *C* was 66. We computed the f-ORG amplitude from these signals using Fourier transform (for separate frequency measurements) or short-time Fourier transform (for chirped stimuli). Averaging multiple measurements yielded a set of six frequency characteristics (one for each subject).

To determine whether the photopic f-ORG cone response also can be decomposed into a sum of two components in the frequency domain as with the single-pulse dark-adapted ORG (37), the data were fitted with the weighted sum of two exponential functions (Fig. 3*B*). This fit matched the experimental results, which was impossible with a single exponential model. The resulting components, referred to as the fast photopic component and the slow photopic component, are plotted in log scale in Fig. 3*C*. The −10 dB cut-off frequencies were calculated, obtaining values of 2.9 Hz and 24.6 Hz for the slow and fast components, respectively. For further comparison, −3 dB cut-off frequencies were also computed. The values were 0.4 Hz for the slow component and 3.7 Hz for the fast component, corresponding to 398 ms and 43 ms time constants, respectively.

**Fig. 4.**
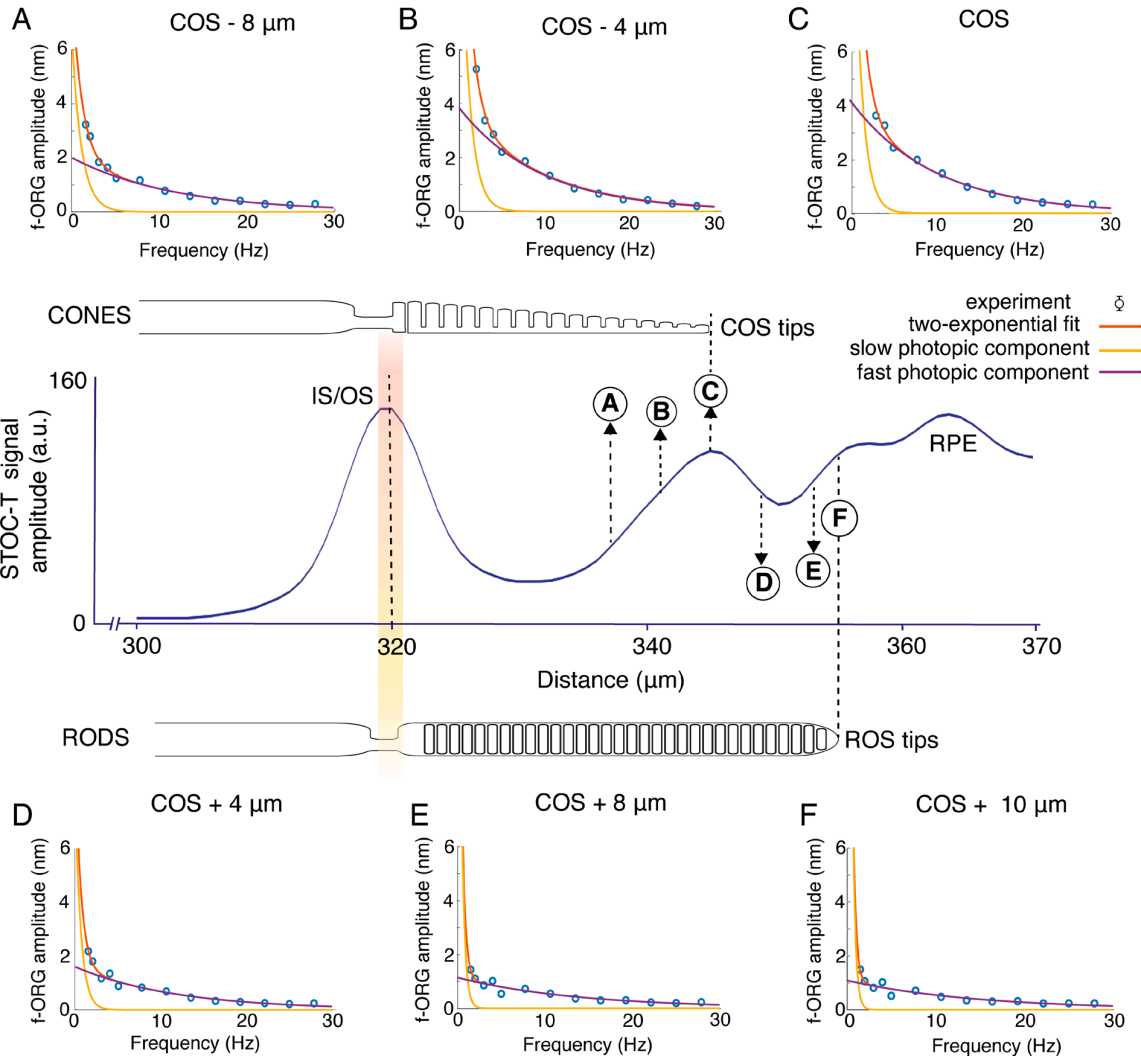
Photopic rod-mediated f-ORG in the human retina in vivo. Variability of f-ORG frequency response for different axial locations in the POS. The amplitude of fast f-ORG responses of ROST is three times smaller than responses detected for COST, with significantly smaller bandwidth. The phase analysis localizations are marked on the outer-segment profile (*A*–*F*) of a representative STOC-T A-scan from one subject (*Middle* panel).

We also analyzed data collected from volunteers to see how the f-ORG signal changes at different positions of the outer segment of retinal cones. This involved measuring the phase difference between IS/OS and various depths around COST and ROST. The STOC-T images (Fig. 2) reveal a spatial separation of rod and cone tips, visible as two distinct layers (with the ROS-tips layer blending into the RPE due to the system’s limited axial resolution which is equal to 5 μm). This separation allows us to analyze the responses of both cones and rods. The frequency-response characteristics of the photoreceptors provide simultaneous insights into the differences in bandwidth responses for cones and rods. As expected, shifting toward the rod-dominated signal (closer to the RPE) reduces the frequency bandwidth to approximately 16 Hz, while the center of the COS layer shows a bandwidth of 24 Hz. The extracted depth-dependent frequency characteristics are presented in Fig. 4 *A*–*F*, with the phase analysis localizations marked on the outer-segment profile of a representative STOC-T A-scan from one subject. It can be observed that as the number of cone photoreceptor discs contributing to the f-ORG signal decreases, the response amplitude of both components decreases (Fig. 4 *A*–*C*). The f-ORG signal magnitude detected from ROS (Fig. 4 *D*–*F*) is about three times smaller than that from COS and decreases as it approaches the area where ROS interdigitate with RPE processes.

### Sildenafil-Affected Rod-Mediated ORG in the Mouse Eye Imaged In Vivo.

**Fig. 5.**
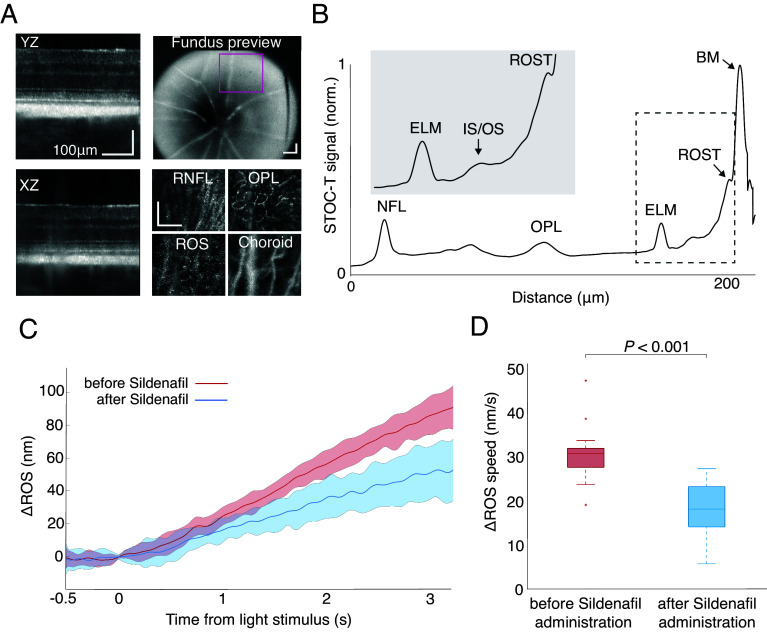
Scotopic single-pulse ORG in the mouse eye in vivo. Effects of sildenafil administration on rod length changes in response to light stimulation. (*A*) Examples of STOC-T images of the retina of a BALB/c albino mouse, along with the preview from a fundus camera. (*B*) STOC-T averaged A-scan acquired before sildenafil administration in scotopic conditions; the *Inset* shows enlarged region covering ELM, IS/OS, and ROST. (*C*) Rod-mediated, single-pulse, scotopic ORG recordings before (red) and after (blue) sildenafil administration. The centerline represents the mean and the shaded area represents ± one SD. (*D*) Boxplot showing the difference in the linear component of the rod elongation velocity in response to a single flash before (red) and after (blue) sildenafil administration. The central mark indicates the median, and the bottom and top edges of the box indicate the 25th and 75th percentiles, respectively. The whiskers extend to the most extreme data points not considered outliers, which are plotted individually. A Welch’s *t* test, or unequal variances *t* test reveals a statistically significant difference in the scotopic ORG response before and after sildenafil administration (*P* < 0.001).

To understand the photoreceptor response to flashing stimuli and identify the processes responsible for changes in photoreceptor length, we performed controlled experiments using FDA-approved sildenafil, a known inhibitor of PDE6 (42–45), a key structural and functional protein in the photoreceptors. Due to the need for robust and durable inhibition of PDE6, we used BALB/c mice in our study, as doses of sildenafil above human safety limits were necessary [toxic for photoreceptors as indicated by Yanoga et al. (46)]. The overdosing of sildenafil was necessary to keep an effective concentration of the inhibitor in the system, as sildenafil is rapidly eliminated in rodents (47–49). We chose the dose to provide a time course of PDE6 inhibition in the mice overlapping with the time of maximum inhibition reported for pigmented (C57BL/6 J) mice, representing normal vision; and for albino BALB/c mice (*SI Appendix*, Fig. S1 *A*-*D*), having slightly worse visually evoked responses in general. Our assessment of the decline in visual response as a function of time was necessary to estimate the appropriate time for the postinjection ORG recording. Although the cyclic elongation and shortening of photoreceptors described above were recorded from human cones, we can interpret the responses from rod-dominant mouse retinas in an analogous manner as both the slow scotopic component and fast photopic response involve the activation (and deactivation) of PDE6 in the phototransduction cascade manifest the same biochemical effect. The ORG data were recorded two to three times from each of the five BALB/c mice (Fig. 5) as they are easier to image but have weaker visual responses than pigmented animals (*SI Appendix*, Fig. S1). The data for each mouse were then averaged and presented as single points in ORG analyses. Representative images of STOC-T cross-sections in B-scan and en-face projections, along with their location and the size of the STOC-T lateral field of view shown in the fundus image, are presented in Fig. 5*A*. When imaging the retina of BALB/c mice, the IS/OS layer and ROS are less prominent than in the human eye (Fig. 5*A*). However, when the 50 A-scans are averaged (Fig. 5*B*), the exact determination of the IS/OS and ROS layers becomes possible, and the relative phase difference after further processing was converted to a nanometric reading. We recorded 77 response datasets before and after the sildenafil administration. Of those, 58 provided good STOC-T image quality, and 48 yielded viable ORG signals with properly suppressed breathing artifacts. The selected ORG responses included 10 from mouse #1, 12 from mouse #2, 11 from mouse #3, 5 from mouse #4, and 10 from mouse #5. With the exception of mouse #4, responses from each mouse were acquired on two nonconsecutive days. The average rod-mediated, single-flash, scotopic ORG response before (red) and after (blue) sildenafil administration was calculated from the 48 datasets (Fig. 5*C*). There is a marked reduction in the response of the photoreceptors after sildenafil administration. The rod-elongation response is flat before the flash-stimulus onset, and mostly linear with time afterward. We evaluated the linear component for each of the 48 rod-elongation responses, excluding 4 curves due to failed linear-component estimation. Fig. 5*D* shows the statistical analysis of rod-elongation linear velocity following the light flash as a boxplot, with median velocities of 31 and 18 nm/s up to a 3-s poststimulus onset, in the absence or presence of sildenafil administration, respectively. A Welch’s *t* test, or unequal variances *t* test, revealed a significant difference in scotopic ORG responses in the absence vs. the presence of sildenafil (*P* < 0.001).

To control photoreceptor activity and observe functional and anatomical changes, we created a pharmacological model of defective photoreceptors using a high dose of sildenafil, an inhibitor of PDE6, and thus a blocker of phototransduction. First, we tested sildenafil’s effect on visually evoked responses in the primary visual cortex (V1) of C57BL/6 J mice, finding a significant drop in amplitude from 198 ± 37 to 27 ± 4.5 µV (*P* < 0.001; *SI Appendix*, Fig. S1 *A* and *B*). An analogous significant drop in V1 amplitude was observed with BALB/c mice (115 ± 21 µV vs. 26 ± 4.2 µV; *P* = 0.002; *SI Appendix*, Fig. S1 *C* and *D*), albeit of somewhat smaller magnitude. Note that BALB/c mice had smaller VEP amplitudes than C57BL/6 J animals in the control condition (198 ± 37 µV vs. 115 ± 21 µV).

**Fig. 6.**
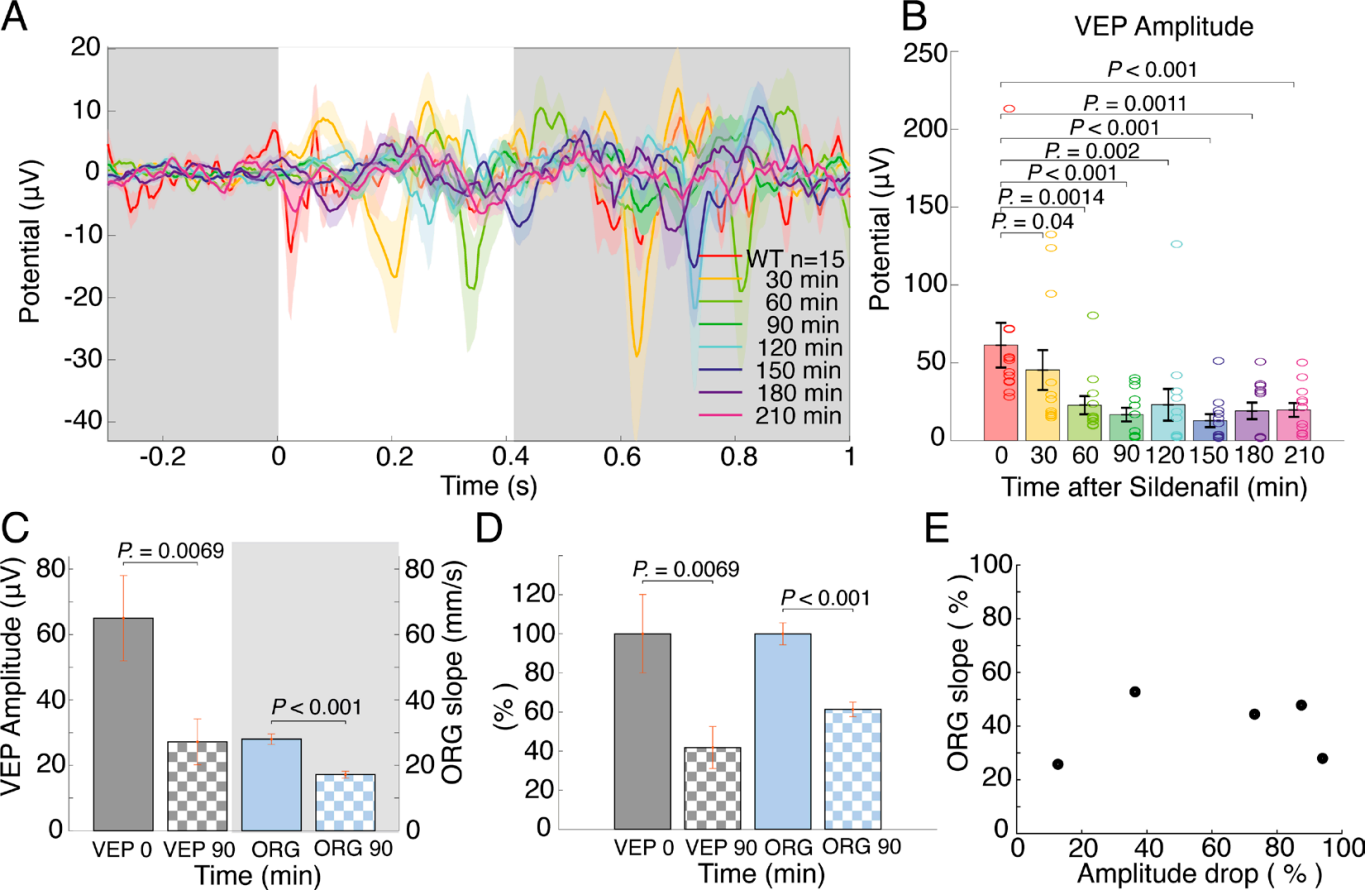
Effect of sildenafil administration on ORG and VEPs in mouse eyes in vivo. (*A*) VEPs recorded in albino animals after ORG sessions, up to 3 h after sildenafil injection. (*B*) Corresponding bar-graph summary of the dynamics of evoked potential responses after sildenafil injection. (*C*) Raw mean amplitudes and slope of the single-pulse, dark-adapted ORG before (0 min) and after (90 min) sildenafil injection. (*D*) Same numbers as in *C*, shown as a percentage of control recordings. (*E*) The scatter plot directly compares the percentage change in the slope of the ORG signal with the decrease in amplitude. Each dot represents a single mouse.

After testing sildenafil’s effect on visual responses under native conditions, we tested the ORG responses before and after sildenafil injection to determine whether inhibiting the phototransduction cascade affects the ORG signal (Fig. 6). To ensure sildenafil’s effectiveness for ORG experiments, we recorded visual responses in V1 in ORG-experiment mice over an extended time. Although BALB/c mice had slightly smaller initial responses, they still showed a significant drop in VEP amplitudes beginning 30 min after sildenafil injection, with a maximum effect (minimum response amplitude) at 90 min [65 ± 14.7 µV (0 min) vs. 16 ± 4 µV (90 min); *P* < 0.001; Fig. 6 *A* and *D*]. The bar graph in Fig. 6*B* shows the collective dynamics of the evoked potential responses we observed after sildenafil injection. Additionally, to check for potential toxicity of the PDE6 inhibition, we examined the thickness of the ONL layer postmortem in ORG-recorded mice. This analysis (*SI Appendix*, Fig. S1 *E* and *F*) revealed only a slight, but not statistically significant, loss of photoreceptors after several high-dose injections of sildenafil, even if the VEP amplitudes were smaller than in the native BALB/c group. Comparing VEP-amplitude changes, before and 90 min after sildenafil injection, to ORG-slope changes revealed significant changes in both (VEP change: *P* = 0.007; ORG slope change: *P* < 0.001) (Fig. 6*C*), which are even more pronounced when viewed as a percentage of control recordings (Fig. 6*D*). Comparing the percent change of VEP amplitude to the percent change of ORG slopes for all five mice showed no direct linear correlation between the two measurements (RHO = 0.18; *P* = 0.78) (Fig. 6*E*).

## Discussion

In this study, we characterized the time course and explored the mechanism for changes in length of the POS in response to single-pulse and flicker-light stimulation. We found that flicker stimulation captures the fast component of the COS response in human retinas under photopic conditions, with time constants matching phototransduction-based cone responses described in the literature (Figs. 2 and 3). Additionally, by simultaneously analyzing the responses of cones and rods at variable stimulation frequencies, we confirmed that their observed elongations are concurrent and likely governed by the same mechanism (Fig. 4). Finally, we demonstrated that the use of sildenafil, which chemically disrupts the phototransduction cascade, also reduces elongation of rods in a live mouse model (Figs. 5 and 6).

Flicker stimulation induced changes in COS length, which were evident by an increase in ∆COS (Fig. 2*D*) over time. This result is similar to that previously observed with single-pulse stimulation (37). The elongation process was split into two different components—fast and slow (Fig. 2 *E* and *F*). With continuous stimulation using flickering light rather than a single pulse, there is a prolonged exposure to the stimulus luminance, resulting in the saturation of only the slow component, which was sensitive to the mean illuminance as if the retina were illuminated by a low-pass filtered version of the stimulus. Additionally, flickering-stimulus experiments revealed an oscillatory component that has a much lower amplitude compared to the overall COS-elongation value (Fig. 2*G*). However, as with any homodyne detection, the signal-to-noise ratio (SNR) can be enhanced using a Fast Fourier Transform (FFT). The average SNR for detecting the 10-Hz oscillatory component was 32 dB across the eight measured ORG responses, as shown in Fig. 2*D*. Notably, the amplitude of the oscillatory signal changes only slightly, from 1.3 to 2 nm, despite a more than tenfold change in peak flicker luminance (0.3 to 3.9 kcd/m^2^) and corresponding bleach levels (1.7 to 18.5 %) (*SI Appendix*, Fig. S2). This result indicates that even when the quality of the delivered stimulus varies significantly (such as in patient measurements), the anticipated response will remain within a consistent amplitude range. This consistency makes the f-ORG method promising for clinical translation. We have previously shown that the average SNR of COS modulation increases with the photon flux applied before flickering (light-adapted case) (33), which is also evident here in the improved quality of the oscillating signal after 1 s from the beginning of stimulation (Fig. 2*E*). Therefore, dark adaptation is not required for f-ORG experiments, reducing measurement time.

In addition to the two elongation components, single-pulse stimulation generates a superfast phase (~5 ms) where COS shrinkage occurs. This phase is explained by changes in the electrical potential and surface tension within photoreceptor disc membranes (35, 37). Using flicker excitation under photopic conditions enables us to saturate the ultrafast and slow components, thereby diminishing the contributions of electrical potential, surface tension of disc membranes, and osmotic swelling.

Photopic flicker ERG is a well-established technique for characterizing the response of the entire neurosensory retina (50). Typically, retinal potential amplitude in photopic ERG with flicker frequency sweep shows a gradual decrease in the 1 to 10 Hz range, followed by a rapid increase to a peak around 30 Hz, and then a subsequent decline between 30 and 60 Hz (50). Our data shown in Fig. 3 do not reflect this pattern. However, our results cannot be directly compared with photopic ERG measurements, as our observations focus on the outer layers of the photoreceptors rather than the entire retinal sensory system. Instead, we can compare our measurements to the measurements of voltage responses to sinusoidal stimuli conducted using the patch-clamp technique in fresh primate retinas (51, 52). The characteristics we obtained closely reproduce the patch-clamp results for L-M cones at illumination levels below 5,000 R*/s (Fig. 3 in ref. 51). In our case, the established cut-off frequencies for a 10% response, approximately 29 Hz for L-M cones, were about 25 Hz. These frequencies depend on excitation intensity and eccentricity (53), which in our experiments did not exactly match the values presented in the Baudin et al. study (51). However, since the values are not significantly different and the characteristics measured in primates and in our experiments are similar, we are confident that the f-ORG signals we observed directly correspond to the local voltage response of cones.

Importantly, similarly to photopic flicker-ERG, photopic flicker-ORG holds a high clinical translational potential as the ability to characterize the frequency response of the photoreceptor modulation length under light-adapted conditions in a matter of few seconds (34) can provide a complementary biomarker of retinal function (or dysfunction). Earlier clinical adoption than other ORG schemes could be favored by the practical benefits of the f-ORG procedure for humans, eliminating the requirements for lengthy measurement times, cumbersome dark-adaptation periods, and a dark clinical room environment.

We were also able to analyze the flicker-light responses of cones and rods recorded simultaneously at various frequencies (Fig. 4). In our experiments, we observed approximately three times less amplitude of the f-ORG signals detected for ROS (Fig. 4 *D*–*F*) than for COS (Fig. 4 *A*–*C*). This result differs from the results obtained by others (54, 55) using a single-pulse stimulus in dark-adapted conditions, where response amplitudes from cones and rods were similar when the light pulse bleached about four times more cone photopigment. The reduction in rod response likely relates to rod saturation due to light adaptation at approximately 175 cd/m^2^ during our experiments (56). Alternatively, the poor sensitivity to phase changes could be due to simultaneous contributions from ROS tips and RPE processes, which strongly scatter light. Another hypothesis suggests that the fast and slow components could be mediated by cones and rods, respectively. If this were the case, however, the slow component amplitude should increase when the signal is extracted from deeper retinal layers closer to the ROS tips than to the COS tips; but the opposite occurs, with the maximum amplitude near the COS tips. Moreover, these results convincingly demonstrate that the observed ORG signals are due to the elongation of the photoreceptors rather than to a change in refractive index (i.e., chemical content), as previously suggested for neuronal cells in the report of Iyer et al. (57). If the changes were due to refractive index variations, the ROS-tips layer and RPE would experience the same or greater phase changes as the COS-tips layer, located closer to the source in the light propagation path. Consequently, the f-ORG signal amplitudes would increase as the analysis window moves toward the RPE. In contrast, our observations show a decreasing amplitude of phase changes, confirming the hypothesis of a geometric change in the length of the POS due to their unique structure. Importantly, the ROS response exhibits a similar elongation phase and matching frequency characteristics (Fig. 4 *D*–*F*), indicating a close correspondence between the responses of cones and rods to flicker stimuli. This result confirmed that further experiments to identify the mechanism responsible for photoreceptor elongation in response to light could be performed on rod-dominant mouse retinas.

In mice, however, we could not perform photopic f-ORG measurements for practical reasons, as the bleaching of rods in photopic conditions led to rod OS-length modulation amplitudes (if any) below the sensitivity of our system, and to an ORG signal confounded by the artifactual influence of vasculature-blood pulsation at frequencies centered around 6 Hz (49). Scotopic f-ORG measurements suffered from similar issues due to prolonged exposure to flickering light. Scotopic single flash experiments on mouse retinas obviated this problem and provided a relevant test for our hypothesis, as we expected the mouse rod OS elongation also to feature contributions by two components, although with longer time constants (26). Therefore, we measured scotopic single-pulse ORG in albino mice to access the phototransduction cascade.

Mouse models in which PDE6 is mutated display photoreceptor degradation (34, 58), which makes them unsuitable for directly testing the role of PDE6 in morphological changes observed via ORG. Therefore, to test the potential role of PDE6 in propagating the responses that we observed, we employed pharmacological inhibition of PDE6 using sildenafil, coupled with measurements of VEP in the mice (Fig. 6). Retinal toxicity in humans is a known side effect of the usage of sildenafil, but we do not expect it to influence our ORG measurements on mice, as the reported cases in humans may arise from other factors unrelated to photoreceptor morphology, such as cGMP imbalance (59, 60), and such toxicity may take hours to be notable, even after overdosing (61). Under our experimental conditions, VEPs showed a continuous decrease as a function of time after sildenafil injection until 90 min (Fig. 6*B*), with relatively flat ORG responses over the duration of each measurement (3 s). The post-sildenafil measurements showed a clear decrease in ROS elongation (Fig. 5*C*) and slower elongation velocity (Fig. 5*D*). There are residual ΔROS changes on scotopic ORG recordings (Fig. 5*C*), which we attribute to the sum of slow and fast components of expansion due to incomplete inhibition of the phototransduction cascade (Fig. 6), further exacerbated by the fact that PDE6 inhibitors like sildenafil paradoxically potentiate cGMP hydrolysis by nonactivated PDE6 (62). Also, we rule out potential osmotic changes in the ROS due to the presence of sildenafil, for compelling reasons: i) other solutes at higher concentrations eclipse the sildenafil concentrations within the ROS (63, 64) and ii) osmotic changes in the ROS are driven by dilation of the choroidal and retinal vessels (65, 66), which sandwich the photoreceptors in the retinal layers; so inhibition by sildenafil of PDE5 in those vessels (45), would expand those vessels and contract the outer-segment layer, rather than expand it, as we report here. Thus, we conclude that the observed ORG changes are a result of the direct effect of inhibition of PDE6 by binding of sildenafil. A third aspect of sildenafil that may lead to the visual impairment reported, is the inhibition of PDE5 in bipolar and ganglion cells (67), but this effect also occurs at a later time after the photoreceptor changes, we detect in this workbut this effect occurs for later reading times of the VEP signals than the photoreceptor changes we detect in this work. The transient and repeatable effects are evident, with nonconsecutive-day measurements on the same mice producing consistent data points within 1 SD (shaded areas) of previous recordings. Plotting VEP amplitudes and ORG slopes before and 90 min after sildenafil injection (Fig. 6*C*) reveals similar patterns after normalization (Fig. 6*D*), despite variations in ORG responses among different mice (Fig. 6*E*).

PDE6 is one of the largest proteins in the COS and ROS, and it is considered to be an excellent candidate for the role of a pillar protein. The PDE6αβ heterodimer forms a pseudo-twofold symmetry attributed to the N-terminal pony-tail helical structure (Pt-motif) between the PDE6α and PDE6β subunits (68). PDE6 has a conserved catalytic phosphohydrolase domain, regulated by GAF domains and PDE6γ-mediated mechanisms (69–71). During phototransduction, PDE6 undergoes conformational changes associated with its allosteric activation (68). This activation occurs when the transducin subunit Gtα-GTP forms a complex with PDE6. Upon light activation, PDE6 elongates by about 30 Å (72). These light-induced conformational changes in PDE6 increase ROS intradiscal spacing to approximately 17 nm due to the rotation of the catalytic domains around an axis parallel to the lamellar membranes. A prolonged light stimulus at full saturation is expected to increase the ROS length from 23.8 ± 1.0 μm (dark) to 26.23 ± 1.1 μm (light), with activation occurring within milliseconds (73). It is likely that cones undergo similar changes.

In humans, the ultrafast contraction of the COS is about 50 nm at most, and the slow expansion is about 200 to 300 nm (21, 25, 35), though it can be up to about 500 nm (21). Without the stacked arrangement of discs in the photoreceptors, the changes observed could not be attributed solely to conformational shifts in a protein the size of PDE6. However, the layered structure of these discs allows for the accumulation of numerous small changes, making such alterations possible. Furthermore, biological data (24, 74) indicate that the lack of a Tα subunit in the phototransduction pathway precludes the expansion of the photoreceptor. As Tα interacts with PDE6, causing conformational changes and activating it, it is highly likely that these conformational changes in PDE6 ultimately drive the morphological changes in the photoreceptors. This can be achieved because the PDE6 catalytic subunits are anchored to the photoreceptor disc membranes via isoprenylation of their C termini (in rods, farnesylated PDE6α and geranylgeranylated PDE6β) (74); and via their N termini (68). Depletion of such C-terminal modifications in cones results in decreased sensitivity to light (and delayed response), together with changes in disc density and overall length of the cones (75). The Pt motif at the N terminus provides structural stability to the heterodimeric PDE6 molecules by forming extensive dimerization interfaces (68), and this motif shows high levels of protein-sequence conservation between different PDE6 subunits (74, 76). Thus, each PDE6 molecule may bridge two discs in the COS or ROS via interactions involving its C and N termini, and induce morphological changes in photoreceptors through its conformational changes.

**Fig. 7.**
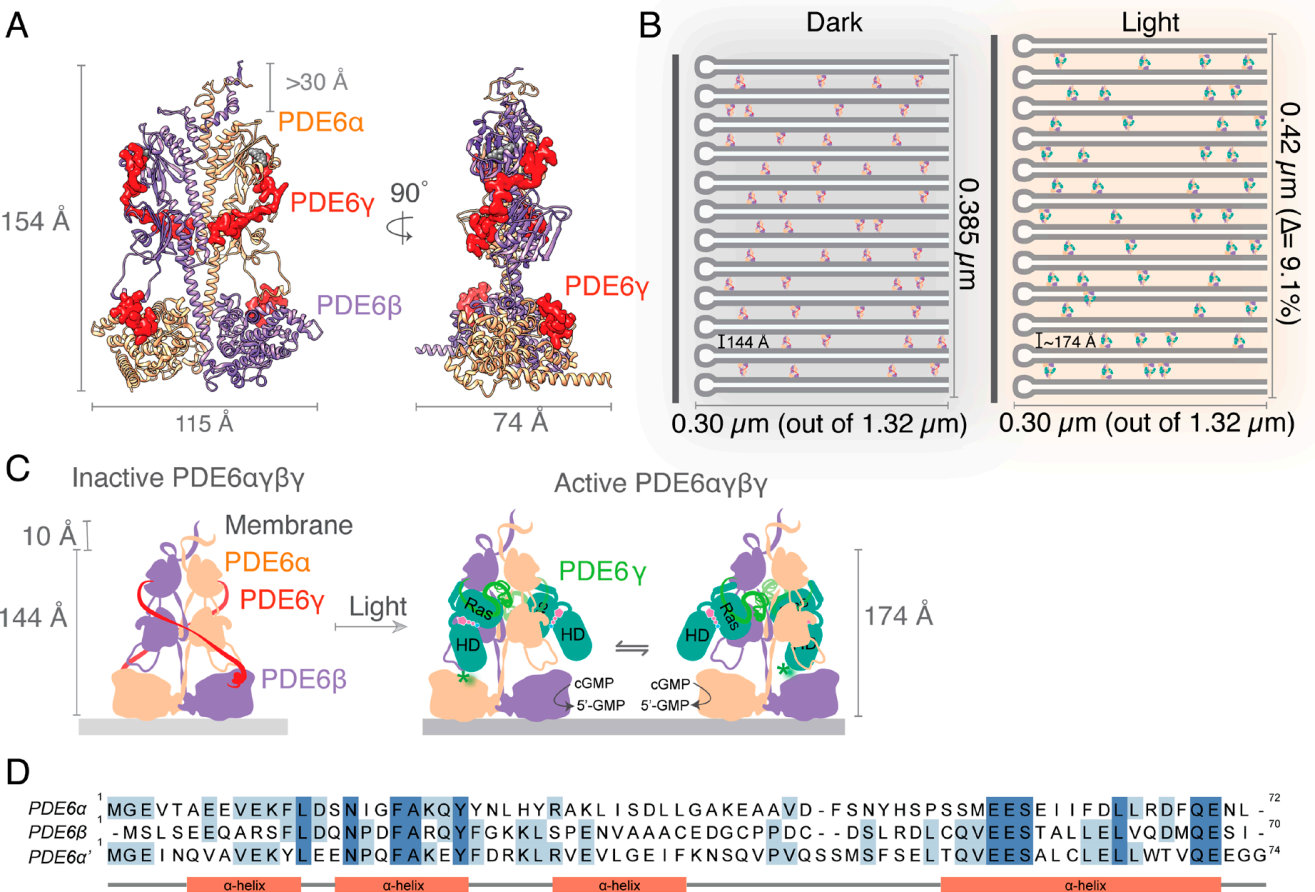
Structural model of PDE6-guided change in ROS morphology. (*A*) Structure of the PDE6 heterotetramer in the ROS, displaying PDE6α (orange), PDE6β (violet), and two molecules of PDE6γ (light red). Two different views (90° apart) are presented. (*B*) Schematic diagram of the potential ROS extension driven by conformational changes of PDE6. Light-adapted ROS have extended interdisc distances of ~174 Å, compared to the 144 Å separation observed in dark conditions (no changes are observed for the intradisc spacing). (*C*) PDE6γ displacement and conformational changes in the catalytic subunits of PDE6 induced by activation of transducin (teal color, with individual domains named). The model of active PDE6 represents the equilibrium between the single activation of α or β subunits. (*D*) Sequence alignment of the PDE6 Pt-motifs for rod- and cone-PDE6 (note: homodimer of α′ subunits). Strictly conserved residues are highlighted in dark blue and partial conservation with light blue. Modified from ref. 72, with permission from Elsevier.

Upon light stimulation, propagated through the phototransduction pathway, G protein transducin T_αGTP_ activates the membrane-attached PDE6, a 3′,5′-cyclic nucleotide phosphodiesterase (62, 77). The membrane colocalization of T_αGTP_ and PDE6 creates a high local concentration of proteins and increases the diffusional encounter frequency (62). Rod PDE6 is a holoenzyme located on the surfaces of the photoreceptor disc membranes (Fig. 7 *A* and *B*). It is composed of two nonidentical catalytic subunits (α and β, ~100 kDa each) and two identical regulatory subunits (2γ, ~10 kDa each) (Fig. 7*A*) (62, 78). PDE6 α β γ_2_ is activated when T_αGTP_ binds, causing a conformational rearrangement of the γ-regulatory subunits, which then exposes the catalytic domain. This activation is regulated by the rate at which cGMP is degraded to GMP (79).

Recent advances in cryoelectron tomography have enabled unprecedented access to the membrane organization of the ROS and confirmed the existence of molecular connectors/spacers between discs that likely contribute to the nanometer-scale precise disc stacking (80), and drive the morphological changes upon light stimulation. Several research groups have attempted to acquire high-resolution structures of PDE6 to elucidate its mechanism of activation, but these efforts have so far been hampered by a lack of inclusion of the native membrane environment (68, 78, 81). The abundance and dimensions of PDE6 suggest that it acts as a “connector” between consecutive discs, as observed in the ROS tomograms (80), and thus serves as the molecular driver of the light-induced morphological changes (Fig. 7*C*). A key element to understanding the scale of these effects, in addition to the stacking of discs, is the amplification mechanism employed in the phototransduction cascade (80). While the exact numbers vary and remain a topic of debate, a significant amplification of the signal is widely recognized as necessary for the effective function of the photoreceptor, from photon detection to cGMP pool depletion.

For each rhodopsin molecule activated by a photon, around 30 transducin molecules are activated, and each of these can, in turn, activate approximately 100 PDE6 proteins. As a result, a single rhodopsin activation can activate up to 3,000 PDE6 proteins, despite only 250 being present on each disc surface. This excess activation potential may influence neighboring interdisc spaces through cross-talk. The amplification (from rhodopsin to transducin to PDE6) observed in phototransduction helps explain the spread-out effect within the photoreceptor and suggests a domino effect of photoinduced changes on the distances between adjacent discs.

Existing literature has indicated that observed ORG signals are related to the transduction process, particularly due to the involvement of PDE6. In the work of Pandiyan et al. (33), the proposed phototransduction model suggests that the fast component of ORG signals corresponds to the free phosphate produced by RGS9-catalyzed hydrolysis of GTP in the α-subunit of the PDE6-complexed G protein. Phototransduction has also been indirectly linked to changes in solute concentration within the cell and the corresponding osmotic balance (24, 37), which is responsible for the second, slower component of ORG signals. As shown in the work of Zhang et al., this component is likely the result of residual opsin activity and the swelling of the disc membrane initiated by photoactivation (24). Our model suggests PDE6 bridges consecutive discs in the POS and, upon activation-induced conformational changes, drives morphological changes along the long axis, not the cross-section (Fig. 7 *B* and *C*). Moreover, the presence of sildenafil in the outer segments is not expected to change its optical properties or the osmotic balances in any other way except from its direct interactions with PDE6, and the resulting inhibition of the phototransduction cascade. Importantly, our model explains the observed interdisc changes and why intradisc distances remain constant upon light stimulation. However, further details of PDE6 membrane attachment via the Pt-motifs at the N termini need clarification. Parallels with other PDEs and strong sequence conservation between cone and rod subunits (Fig. 7*D*) suggest helical and amphipathic properties supporting membrane binding of the N termini of the catalytic subunits.

Conformational changes in PDE6 structure linked to its function in phototransduction, its attachment to disc membranes, and its tenable bridging of consecutive discs in POS have been reported previously. Here, using in vivo scotopic ORG measurements on mouse eyes treated with sildenafil, we provide further evidence implicating PDE6 structural flexibility in mediating the observed morphological changes in photoreceptors upon light stimulation for both slow and fast time responses. Definitive high-resolution structural biology studies will be required to establish how such changes in PDE6 conformation are connected to changes in outer segment morphology during the visual cycle. We also show that the fast cone response observed as an isolated signal in human photopic f-ORG measurements corresponds to the frequency characteristics of the cone electrical response for phototransduction in primates. These findings pave the way for the educated use of ORG measurements as a diagnostic tool, correlating observed biomarkers (i.e., optical path length changes) directly with a molecular driver within the phototransduction pathway. This approach will also have value in tracking other components of the phototransduction cascade, and in facilitating therapeutic evaluations, such as gene therapy for LCA-1 patients with photoreceptor retGC1 disorders. Our work also demonstrates the potential to perform measurements without preadaptation to darkness, thereby enhancing patient comfort, standardizing diagnostic protocols, and reducing functional measurement times—a crucial factor in busy ophthalmic practices. Early symptom detection and monitoring of new therapies, including gene, regenerative, and interventional treatments, could be significantly enhanced by f-ORG, promoting rapid adoption by clinicians and patients.

## Materials and Methods

### STOC-T Imaging Systems Were Built and Used to Assess Structural Changes in Human and Mouse Photoreceptors.

Sildenafil drug treatments were performed to inhibit the transformation of PDE6 into its active conformation. Statistical significance was determined by Welch’s *t* test, or unequal variances *t* test one- or two-way (ANOVA), *P* values < 0.05 were considered statistically significant. Additional details are available in *SI Appendix*.

### Ethical Statement.

#### Human.

The study protocol was approved by the Ethics Committee of the Collegium Medicum of Nicolaus Copernicus University, Bydgoszcz, Poland (KB 87/2021) in accordance with the Helsinki Declaration. All volunteers provided written informed consent.

#### Mice.

All animal experiments were conducted following the European Union directive on laboratory animal use (2010/63/EU) to minimize pain and suffering and were approved by the Local Ethical Committee in Warsaw (1401P/2022). C57BL/6 J and BALB/c were bred in the animal facility of the Nencki Institute of Polish Academy of Sciences. Mice were initially anesthetized with 3% isoflurane (Virbac, Carros, France) in an induction chamber and then placed in a custom-made stereotactic platform to position the eye for optimal imaging. Body temperature was maintained with a custom-made heating pad in the platform. Before recordings, eyes were treated with Neosynephrin-POS 10% (phenylephrine 100 mg/mL) and Tropicamidum WZF 1% (tropicamide 10 mg/mL). During STOC-T recordings, BALB/c mice were kept under 1% isoflurane anesthesia, with eyes covered in protective gel and contact lenses. After data collection, mice were returned to their cages and placed on a heating pad for recovery.

#### Scotopic and Photopic f-ORG Method for the Human Eye In Vivo.

##### STOC-T imaging system.

The STOC-T method is a new imaging modality combining the principles of OCT, optical diffraction tomography (ODT) (82), and digital holographic microscopy (DHM) (83). STOC-T is capable of capturing three-dimensional images in vivo at unprecedented high speeds without the adverse effects caused by strong light scattering in living tissue (30, 84–87). STOC-T uses controlled spatial coherence of light by generating hundreds of unique patterns of beam illuminating the sample (so-called transverse electromagnetic modes) that enter a dedicated optical system at intervals longer than the total coherence time of the light source as defined by its spectral width. As a result, the STOC-T system produces several hundred sets of uncorrelated digital holograms, one after another, for each optical wavelength illuminating the imaged object. These holograms are averaged during camera integration and thus signals from undistorted images are amplified and signals from distorted images are attenuated. High imaging quality is achieved by preserving the phase relationship with respect to a flat reference mirror, and maintenance of this phase relationship is the basis for undisturbed images (84, 85). Partial spatial coherence adds a new dimension to imaging, necessitating extra signal processing steps beyond those used in standard OCT. These steps include applying additional 2D FFT filtering to holograms captured at varying wavelengths, as well as performing digital corrections for defocus and aberrations. The STOC-T technique was previously presented in a publication by Auksorius et al. (30). This technique offers rapid registration of volumetric retinal information within 5 ms per volume (256 × 256 × 256 pixels resulting from an acquired 512 frames for variable optical frequency of the illuminating laser) of the human retina, reducing the effect of optical crosstalk (30). This enables high-contrast retinal reconstruction with isotropic resolution of 5 µm in all three dimensions, achieved by numerically correcting the eye’s optical aberrations (86). The STOC-T device employs a Michelson interferometer with a swept-source laser (Superlum, Broadsweeper BS-840-2-HP), which sweeps linearly in wavenumber from 803 to 878 nm (*SI Appendix*, Fig. S3). The light source is connected to the interferometer by a long multimode optical fiber serving as a spatial phase modulator (30, 31, 33). The power at the cornea is 3.4 mW measured at 840 nm. Combined with an ultrafast camera (Photron, SA-Z) and full-field detection, the system can achieve a high volume rate of ~188 vol/s for 256 × 256 × 256 or ~112 vol/s for 512 × 512 × 256 processed voxels. For structural imaging, the system operates at 60,000 frames per second (fps), providing a volume rate of 112 Hz. Similar to other interferometric techniques, STOC-T enables the detection of phase changes in the interferometric fringe signal with an accuracy that is four orders of magnitude greater than the nominal axial resolution of the imaging system. This precision allows for the measurement of subnanometer variations in the structure being analyzed (88). During ORG measurements, the system’s speed was set to 100,000 fps and volume size to 256 × 256 × 256, resulting in a volume rate of 188 Hz. For low-frequency functional ORG (f-ORG) measurements, the volume rate is reduced to 47 Hz by increasing the time interval between the acquisition of consecutive volumes. A key component of the ORG-measurement setup is the stimulation path, which includes a white LED, a set of spectral filters (450, 520, and 630 nm), and a digital micromirror device (DMD). The DMD, illuminated by the LED and conjugated to the retinal plane, facilitates spatial shaping of the stimulation pattern. Temporal shaping is achieved by modulating the LED current. The measured rise and fall times were below 3 ms, shorter than the acquisition time of a single frame, enabling us to neglect their effect on the flicker.

##### Scotopic f-ORG.

Initial eye imaging involved setting the system parameters to ensure sharp contours of the retinal vessels in structural images. The eye then underwent a further 5-min dark adaptation. After reassessing and fine-tuning the eye’s position to the desired configuration, measurements commenced. The flicker stimulation started after the acquisition of the first OCT volume at 10 Hz, transitioning from dark to maximum illuminance. To address issues associated with harmonics of square modulation, a sinusoidal signal was employed to drive the LED current and modulate the LED intensity (34). During measurements, 340 volumes were collected at a rate of 188 Hz, covering an area of 0.42 × 0.42 mm^2^ of the retina. Measurements were performed three times for each luminance value with a 5-min dark adaptation period preceding each run. ORG signals were obtained by processing STOC-T spectral volumes using our previously described approach (33, 34). The raw f-ORG signal, representing phase changes between IS/OS and COST, was extracted from the complex representation of STOC-T structural signals, using 10 μm thick slab (five pixels in depth after zero padding) for each layer. Radian values were converted to optical path length expressed in nanometers using the formula: $f-\mathrm{ORG}=\frac{\Delta \theta }{4\pi }\lambda_{0}$ (21), where Δθ is the measured phase change and λ_0_ is the central wavelength of the OCT beam. Structural retina images were processed using a pipeline with a numerical aberration correction, as detailed in our previous work (30). To capture both slow and fast cumulative dark-adapted responses to flicker, the raw ORG signal was decomposed into two exponential components using Matlab’s “exp2” model ($ae^{\mathrm{bx}}+ce^{\mathrm{dx}}$). For response plots, the appropriate fit component ($ae^{\mathrm{bx}}$ or $ce^{\mathrm{dx}}$) was subtracted from the raw ORG signal. Remaining flicker oscillations were presented by subtracting both fit components and by high-pass filtering the signal with a cut-off frequency of 2 Hz. The given bleach values were computed using Psychophysics Toolbox from trolands. The troland values were calculated based on the stimulation spectrum, the radiant power measured at the cornea, and the size of the stimulation beam on the retina, in accordance with the procedure outlined in the literature (89).

##### Photopic f-ORG.

The experimental protocol begins with initial eye imaging, setting system parameters to ensure sharp contours of retinal vessels in structural images. After securing proper positioning, the subject’s eye is dark-adapted for 3 min, followed by a 1-min light adaptation period. This initial dark adaptation ensures comparable conditions for each measurement, and the light adaptation establishes a stationary retina state that will be perturbed by the flickering stimulation. Although a shorter light adaptation period could be sufficient, a 1-min duration accommodates practical considerations such as position adjustments and suppression of eye pupil vignetting of the retinal image.

After preparation, the measurement process commences with configurations depending on the bandwidth of interest. For full photoreceptor frequency-response characterization, measurements were divided into two frequency bands. For 1.5 to 5 Hz, the f-ORG signal was acquired with single-frequency stimulation (33) at 1.5, 2, 3, 4, and 5 Hz (starting at 1.5 or 2 Hz, depending on the subject), collecting 340 volumes at 47-Hz volume rate and covering an area of 0.42 × 0.42 mm^2^. Considering the signal processing pipeline, flicker initiation coincided with the acquisition of the first volume (no baseline acquisition). Each frequency measurement was repeated three times. For frequencies above 5 Hz, a linear chirp in flicker stimulation ranged from 5 to 45 Hz, collecting 340 volumes at 188 Hz in a frame size of 256 × 256 pixels. Each measurement was repeated at least three times. RAM limitations of the Photron camera constrained the volume number and measurement resolution, though different equipment could improve these parameters. Photopic f-ORG signals were obtained by processing STOC-T spectral volumes using the previously described approach (33, 34). The f-ORG amplitudes were computed in two ways. For low-frequency measurements (1.5 to 5 Hz), f-ORG amplitudes were obtained one at a time for each excitation frequency by reading the maximum value from the amplitude spectra of the band-pass-filtered f-ORG signals (33). For higher frequencies (5 to 45 Hz) the amplitudes were obtained from the spectrograms of high-pass-filtered chirped f-ORG signals (34). The combined characteristics formed a response across a wide frequency range, later decomposed into two exponentials, using the Matlab exp2 model for slow and fast light-adapted f-ORG components as seen in the dark-adapted f-ORG case. For Fig. 4, the thickness of the layers used for ORG signal extraction was reduced from 5 pixels to 3 pixels in depth, to allow for improved tracking of signal changes with depth. When computing the cut-off frequencies at the ROST position, the data range used for fitting was limited to 1.5 to 15 Hz due to low SNR at higher frequencies in this layer.

## Supplementary Material

Appendix 01 (PDF)

## Data Availability

All data are included in the manuscript and/or *SI Appendix*.
