# Supplementary material for: Photopic flicker optoretinography captures the light-driven length modulation of photoreceptors during phototransduction
Source: Proc Natl Acad Sci U S A. 2025 Feb 13;122(7):e2421722122. doi: 10.1073/pnas.2421722122 (PMC11848411; doi:10.1073/pnas.2421722122)
Supplement: Supplementary file 1 — Appendix 01 (PDF) [file pnas.2421722122.sapp.pdf]

PNAS

Supporting Information for

Photopic flicker optoretinography captures the light-driven length modulation of photoreceptors during phototransduction

Sławomir Tomczewski[a,b], Andrea Curatolo[a,b,c], Andrzej Foik[a,b], Piotr Węgrzyn[a,b,d], Bartłomiej Bałamut[a,b], Maciej Wielgo[a,b], Wiktor Kulesza[a,b], Anna Galińska[a,b], Katarzyna Kordecka[a,b], Sahil Gulati[e], Humberto Fernandes[a,b], Krzysztof Palczewski[f,g,h,I,1], Maciej Wojtkowski [a,b,1]

[a]International Centre for Translational Eye Research, Skierniewicka 10a, 01-230 Warsaw, Poland

[b]Institute of Physical Chemistry, Polish Academy of Sciences, Kasprzaka 44/52, 01-224 Warsaw, Poland

[c]Department of Physics, Politecnico di Milano, Piazza Leonardo da Vinci, 32, 20-133 Milan, Italy

[d]Faculty of Physics, University of Warsaw, Pasteura 5, 02 - 093 Warsaw, Poland

[e]Gatan, Inc., Pleasanton, CA, 94588.

[f]Center for Translational Vision Research, Department of Ophthalmology, Gavin Herbert Eye Institute, University of California, Irvine, Irvine, CA 92697, USA;

[g]Department of Physiology and Biophysics, University of California, Irvine, Irvine, CA 92697, USA;

[h]Department of Chemistry, University of California, Irvine, Irvine, CA 92697, USA;

[i]Department of Molecular Biology and Biochemistry, University of California, Irvine, Irvine, CA 92697, USA

[1]To whom correspondence may be addressed. Email: mwojtkowski@ichf.edu.pl, kpalczew@uci.edu

This PDF file includes:

Supporting text
Figures S1 to S5

METHODS

Scotopic Single Pulse ORG of the Mouse Retina *In Vivo*

STOC-T Imaging and ORG Recordings: The optoretinography system for mice, including a STOC-T system, light-stimulation channel, and IR-fundus imaging system for focal plane adjustment, has been described in our previous work (90), and is shown in **Fig. S4**. The STOC-T system performed a laser sweep from 803 to 878 nm linearly in wavenumber units, while the ultrafast camera (Photron, Nova S-16) recorded 512 images at 384 x 256 pixels at a rate of 100, 000 fps within 5.12 ms. The inter-volume delay was set to 7.68 ms, acquiring 340 volumes over 4.352 s (40 volumes pre-stimulus, 300 volumes post-stimulus). A green LED (M530L3, Thorlabs) served as the stimulus, projected onto the retina in a roughly collimated 1 mm^2 spot, consisting of a single 1 ms flash with 125 μW of power. Potential photothermal and photochemical damage was initially estimated based on the maximum permissible exposure (MPE) for human eyes, scaled to the relative volume or numerical aperture of mouse eyes, with retina functionality verified post-measurement using electrophysiological tests. An accurate 6-degree-of-freedom mouse positioner with an anesthesia-delivery mechanism and heated bed and temperature control was built (**Fig. S5**). Proper positioning relative to the 0-diopter meniscus lens interface is crucial for successful volumetric acquisition. The lens maintains lubrication and removes variability in refractive power, increasing measurement repeatability (91). An immersion wet lens interface minimizes mismatch in refractive indices, ensuring accurate optical path length control and imaging plane stability. For single-pulse protocol acquisition, mice were dark-adapted for 12 hours before starting the protocol, initiated *via* custom software (LabView) ensuring synchronized control over the STOC-T light source, camera, and stimulus LED timing. The STOC-T acquisition then proceeded as described above. The protocol was repeated after a 5-minute dark adaptation period, three times in total. Sildenafil injection (2.8 mg) required a 30-minute wait before repeating the protocol. Measuring the same animal before the administration of Sildenafil (as a control group) and after the administration of the drug allowed us to present the effect of phototransduction inhibition in animals measured in an identical setting with the STOC-T system, under comparable illumination and anesthesia conditions.

STOC-T Image Reconstruction and Processing: Following previously described methods (30, 31, 33), STOC-T volumes were reconstructed using the following algorithmic sequence: selection of the region of interest in depth (Z), numerical dispersion compensation, fast Fourier transformation along the wavenumber axis (*i.e.*, the time axis), and normalization by the noise floor. Volume flattening was applied within each volume by detecting the depth of Bruch's Membrane (BrM) and co-registering all A-scans to it. Volume co-registration followed, aligning all volumes to the first volume initially in Z to BrM depth, and then in the X,Y plane. To evaluate intra-volume phase differences between the photoreceptor inner/outer segment junction (IS-OS) and the rod

outer segment tips (ROST), sub-volumes containing three en face planes around these depths were calculated. The inter-volume phase difference in time was evaluated by multiplying each complex sub-volume by the complex conjugate of the first sub-volume, and the cross-correlation matrix was also evaluated and later used to filter-out breathing artifacts. Vascular Doppler artifacts were masked based on the absolute value of the complex mean of the intra-volume product in time and depth axes. Additionally, an oval mask excluded vignetted portions in the image periphery. Resulting sub-volumes from intra-layer, inter-time lag products were complex-averaged in all three spatial dimensions (x, y, z), and phase differences were translated into rod outer-segment optical-path length elongation by multiplying by $\frac{\lambda_0}{4\pi}$.

Recordings of Visually Evoked Potentials: C57BL/6J and BALB/c mice were initially anesthetized with 3% isoflurane in O_2, then placed in a stereotaxic apparatus. General anesthetic (Meloxicam – 0.1 mg, Dopharma Research B.V. – Zalmweg, Raamsdonksveer, Netherlands; Butomidor – 0.1 mg every 2 hours, Bioveta, Czech Republic) and local subcutaneous injections of lidocaine (0.5 %) (Lidocaini hydrochloridum 2%, Polfa Warszawa SA, Warsaw, Poland) were administered. A custom-made plastic chamber was secured to the exposed skull using dental acrylic. After one day of recovery, re-anesthetized animals were placed in a custom-made hammock, maintained under isoflurane anesthesia (2% in O_2). A craniotomy was performed, and multiple single tungsten electrodes were inserted into V1 layers II-VI. After electrode placement, the chamber was filled with sterile agar and sealed with sterile wax. Animals were then kept under light isoflurane anesthesia (0.2 – 0.4% in O_2) throughout the recording procedure. EEG was monitored, and body temperature maintained with a custom-made heating pad (92).

Data were acquired using a multi-channel Scout recording system (Ripple, UT, USA). Local field potentials (LFP) from multiple locations at matching cortical depths were band-pass filtered from 0.1 Hz to 250 Hz, and stored along with spiking data at a 1 kHz sampling rate. The LFP signal was aligned to stimulus time stamps and averaged across trials for each recording depth to calculate visually evoked potentials (VEP). Visual stimuli were generated in Matlab (Mathworks, USA) using Psychophysics Toolbox and displayed on a gamma-corrected LCD monitor (Acer Predator, 35 inches, 100 Hz; 93 cd/m² mean luminance, Acer Inc., New Taipei, Taiwan). Stimulus-onset times were corrected for monitor delay using an in-house designed photodiode system. Visual responses were assessed according to previously published methods (93, 94). For visually evoked response recordings, animals were tested with 100 repetitions of a 500-ms bright flash of light (310 cd/m²). After recording a depth profile to assess the strongest response, the first set of 100 flashes was recorded. Sildenafil solution (0.8 ml of 3.5 mg/ml in saline (2.8 mg)) was administered intraperitoneally, followed by consecutive recordings every 30 minutes for 3.5 hours post-injection. The recordings were performed with three sets of animals: 1) C57BL/6J, 2) BALB/c, and 3) BALB/c

after several f-ORG sessions. This approach enabled us to present the effect of phototransduction inhibition in animals of different backgrounds and conditions (**Fig.6AB** and Supplemental **Fig. S1**).

Visually evoked potentials (VEP) were calculated by segmenting the local field-potential signal based on stimulus-onset time marks, and averaging over 100 repetitions per recording site in each animal. Response amplitude was calculated as the potential difference between the most negative and positive components of the VEP. Data from all electrodes and animals were averaged and plotted as bar graphs. Statistical pairwise comparison was done using the nonparametric Wilcoxon test, considering differences to be statistically significant when the p-value was lower than 0.05.

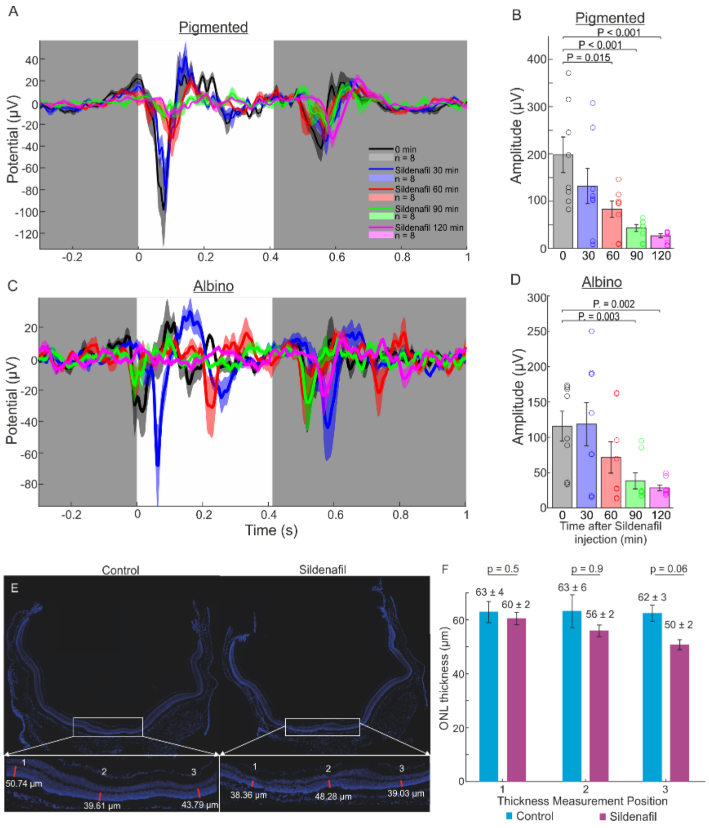

Figure S1. Sildenafil injection dampens visual responses in pigmented, and albino mice. A) Averaged visually evoked potentials (VEP) from the visual cortex of pigmented animals before and 30, 60, 90, and 120 min after the injection of Sildenafil. B) Bar graph and statistics showing the decreases in response after the Sildenafil injection in pigmented mice. C) The VEPs of the albino mice before and after Sildenafil injection, as in A. D) Bar graph presenting average population-response amplitudes decreasing with time after the Sildenafil injection in albino animals. E) Examples of mouse retinal cryosections from control animal and after several Sildenafil injections. Insets show three places used to calculate statistics. F) Bar graph shows the comparison of ONL thickness in control and sildenafil treated animals measured in three spots shown in E.

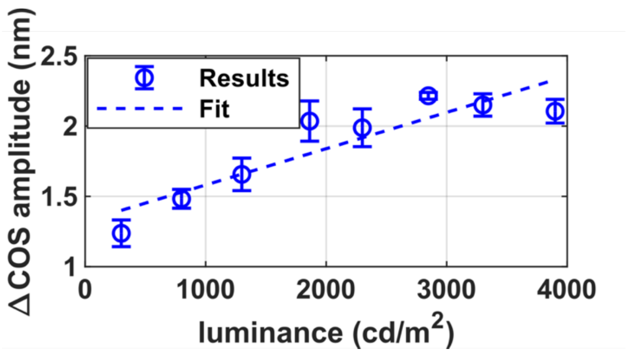

Figure S2. Relation between ΔCOS and Luminance in Dark Adapted f-ORG: Change in the amplitude of the oscillatory response of the cones to a flicker stimulus as a function of stimulus luminance. Fit function parameters (with 95% confidence bounds) for ΔCOS = p1*luminance + p2; p1 = 0.0002; p2 = 1.323.

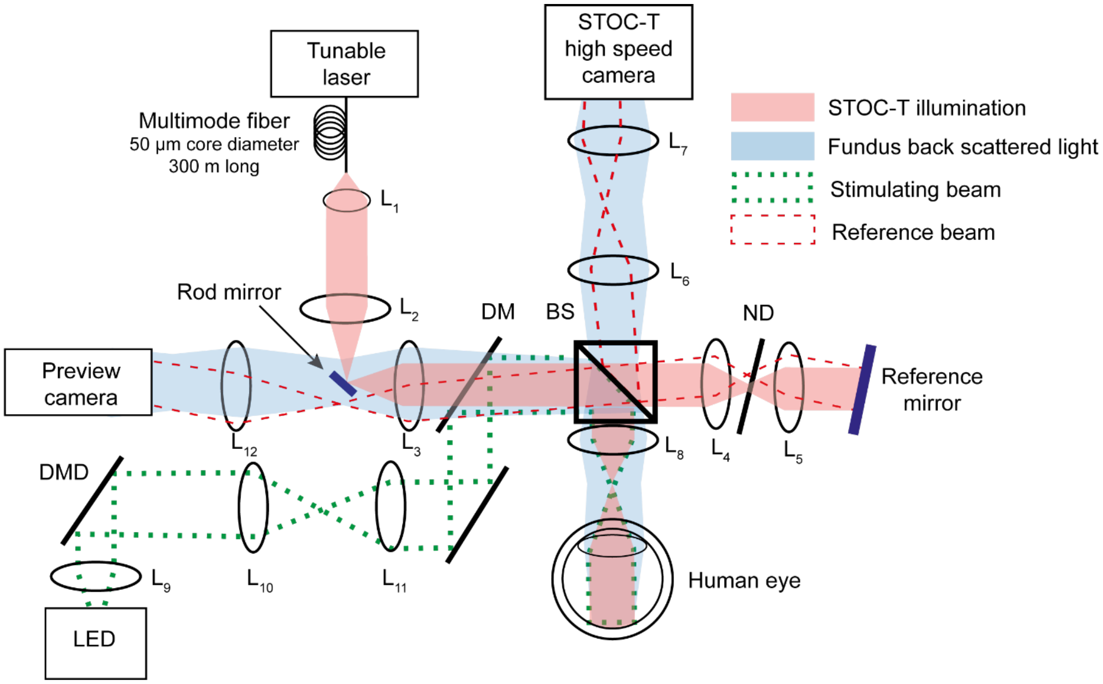

Figure S3. STOC-T f-ORG system for retinal imaging of the human eye. L1-L12, achromatic lenses; DMD, digital micromirror device; DM, dichroic mirror; BS, beam splitter; LED, light-emitting diode. 300 meters of multimode fiber provides effective reduction of optical crosstalk and enables high-contrast imaging of the photoreceptor layer. The reference mirror plane and the retinal plane are conjugated to the high-speed and preview cameras, and the DMD plane is conjugated to the retina. The reference mirror is slightly inclined so that the reference beam is not blocked by the rod mirror. The green dotted lines represent a path for a patterned flickering stimulus.

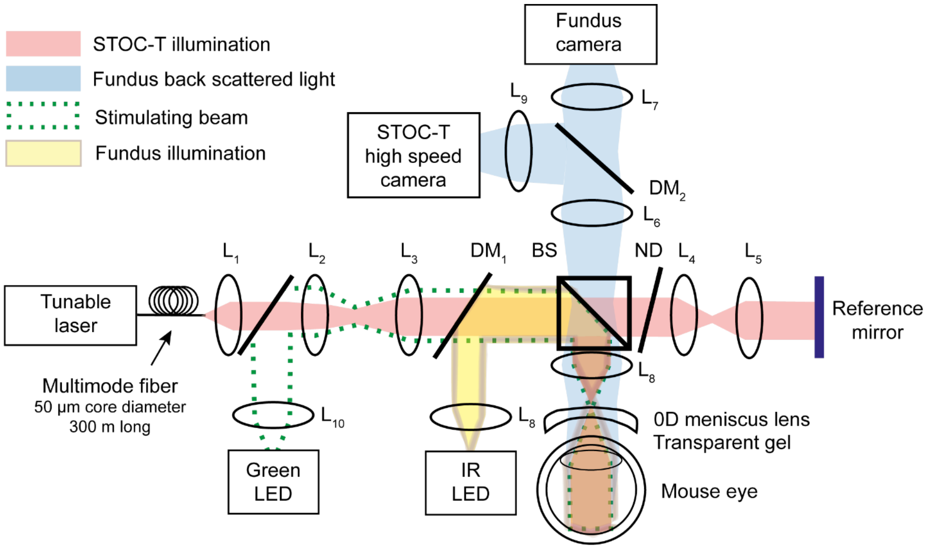

Figure S4. Schematic Diagram of the STOC-T system for mouse imaging. NIR light from the swept-source laser (BS-840-2-HP, Superlum) is delivered to the system through 300 meters of multimode (MM) fiber with a 50 μm core diameter (FG050LGA, Thorlabs). The light is then collimated by an aspheric lens collimator L_1 (APC22-780, Thorlabs), expanded by a telescope consisting of two achromatic doublet lenses, L_2 (f = 30 mm) and L_3 (f = 75 mm), and split into the reference arm and the sample arm using a beam splitter. The optical components in the sample arm include an achromatic doublet lens L_4 (f = 50 mm) and a zero-diopter (0 D) meniscus lens (Cantor & Nissel), with lubricant gel (Vidisic, Bausch & Lomb) serving as the sample interface. The mouse eye is brought into contact with the gel, and the gel thickness is adjusted by repositioning the mouse relative to the meniscus lens. Lens L_4 focuses the image of the MM fiber tip near the front focal plane of the mouse eye, allowing the retina to be illuminated by a low-divergence beam that creates an illumination area on the retina with a diameter of approximately 450 μm. NIR light backscattered by the retina travels back through the eye, L_4, and the beam splitter to form an image of the retina on an intermediate conjugate plane, which is then relayed to the sensor of a high-speed camera (Nova S16, Photron) *via* a 1:1 telescope composed of two achromatic-doublet lenses, L_7 and L_8 (f = 75 mm), in the detection path. An iris is placed in a conjugate plane of the mouse pupil behind L_7 to control the system's aperture, thereby adjusting the lateral resolution and depth of field (DOF). In the reference arm, light is reflected by a silver mirror after passing through an achromatic doublet L_5 (f = 50 mm) and a small plano-convex lens L_6 (f = 2 mm, 2.5 mm aperture), which simulates the focal length of the mouse eye to produce a similar field curvature at the STOC-T camera as that from the sample arm. A neutral density filter is employed to optimize the reference arm power within the STOC-T camera's dynamic range, maximizing sensitivity. For optoretinography, the stimulus source was a green LED (M530L3, Thorlabs) integrated into the STOC-T NIR-illumination path before lens L_2 *via* a dichroic mirror (DMLP650, Thorlabs), following collimation by an achromatic doublet lens (f = 50 mm).

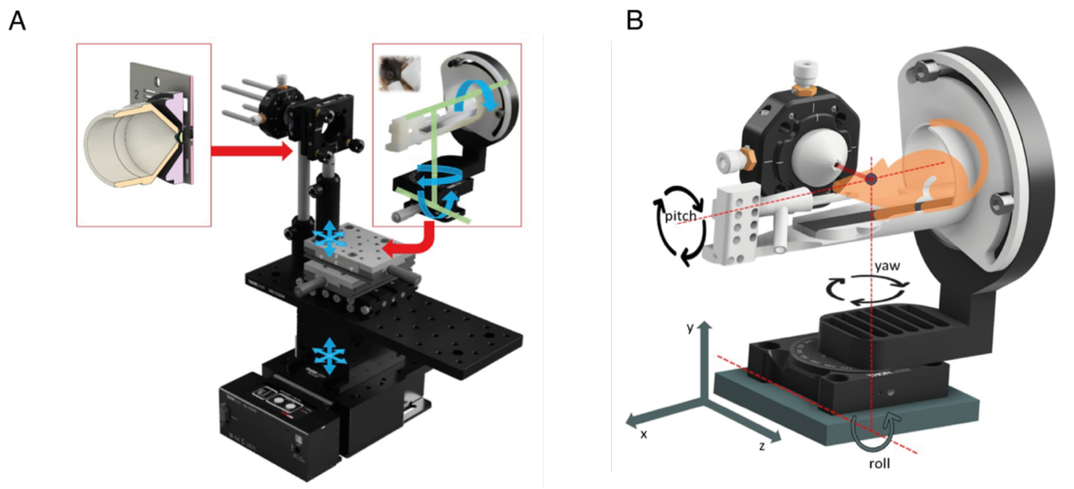

Figure S5. Six-axis mouse holder and manipulator: A. Photograph of the complete platform, with the degrees of freedom indicated. B. Photograph of the mouse holder, illustrating the positioning and orientation of the animal relative to the meniscus lens holder. The mechanical platform enables lateral movement through three motorized steps, while rotation is controlled by three manual mechanical steps. The rotational stages are designed so that the center of rotation aligns with the center of the eye, applicable to both pitch and yaw. The custom-made mouse holder includes an anesthetic-delivery system with a bite bar, and features a heated bed with temperature control.
